# No effect of learning from excellence on patient safety culture in a postanaesthesia care unit: a longitudinal quasi-experimental study

**DOI:** 10.1186/s12913-026-15405-1

**Published:** 2026-08-26

**Authors:** Gørill Birkeli, Owen Matthew Truscott Thomas, Randi Ballangrud, Ellen Catharina Tveter Deilkås, Anne Karin Lindahl

**Affiliations:** 1 Division of Surgery, Akershus University Hospital, Sykehusveien 25, Lørenskog, 1478 Norway; 2 Faculty of Medicine, Department of Health Management and Health Economics, Institute of Health and Society, University of Oslo, Forskningsveien 3A, Oslo, 0373 Norway; 3 Health Services Research Unit, Akershus University Hospital, Sykehusveien 25, Lørenskog, 1478 Norway; 4 Faculty of Medicine and Health Sciences, Department of Health Sciences in Gjøvik, Norwegian University of Science and Technology, Teknologivegen 22, Gjøvik, 2815 Norway; 5 Department of Quality Improvement and Patient Safety, Norwegian Directorate of Health, Vitaminveien 4, Oslo, 0483 Norway

**Keywords:** Organisational culture, Positive psychology, Patient safety, Non-randomised controlled trials, Resilience, Psychological, Learning health system, Formative feedback, Risk management, Postoperative care, Nurses

## Abstract

**Background:**

The prevailing strategy for combating surgically related adverse events (AEs) is to anticipate and prevent them by learning from adverse events (Safety-I). However, not all AEs can be anticipated or prevented in complex systems. This requires more focus on how daily work succeeds, that is, resilient and adaptive behaviour (Safety-II). Learning from success and promoting a positive patient safety culture (PSC) are key global strategies for eliminating preventable AEs. As a patient safety initiative, Learning from Excellence (LfE) captures episodes of successful everyday work and is thus, a system for applying a Safety-II perspective. However, its effect on PSC has not been evaluated. This study aimed to explore whether the implementation of LfE in a postanaesthesia care unit over a 16-month period changed the nurses’ perceptions of the measurable PSC dimensions ‘work engagement’, ‘teamwork climate’, ‘working conditions’ and ‘safety climate’.

**Methods:**

This longitudinal two-group, controlled quasi-experimental study used an annual ‘Hospital Staff Survey’ to measure PSC factors before (2021–2022) and after (2023–2024) the implementation of LfE. The study included nurses from the surgical department of a Norwegian university hospital working in units specialised in postoperative care (intervention unit) and intensive care (comparison unit). The primary outcomes were binarised versions of individuals’ positive responses to Likert-type statements regarding ‘work engagement’, ‘teamwork climate’, ‘working conditions’ and ‘safety climate’. The data were analysed using logistic regression methods.

**Results:**

Relative to the changes in the comparison unit, the results within the intervention unit indicated non-significant small positive changes in ‘work engagement’, ‘teamwork climate’ and ‘working conditions’ and a non-significant small negative change in ‘safety climate’.

**Conclusions:**

LfE did not have a significant effect on PSC after 16 months implementation. Further longitudinal experimental studies are needed to measure the effect of LfE on PSC. Future research should focus on the role of psychological safety in LfE and assess whether reflexive spaces add value to LfE.

**Trial registration:**

ClinicalTrials.gov NCT05794490; registered date: 4 March 2023.

**Supplementary Information:**

The online version contains supplementary material available at https://doi.org/10.1186/s12913-026-15405-1.

## Background

An estimated 313 million annual major surgeries are performed globally, making postoperative care an indispensable component of healthcare [1]. While surgical procedures are meant to save lives, unsafe surgical care can cause considerable harm to patients [2]. In this study, patient harm is termed adverse events (AEs) and is understood as injuries ‘that was caused by medical management (rather than the underlaying disease) and that prolonged the hospitalisation, produced a disability at the time of discharge, or both’ [3] p.370. Surgically related AEs are the second most common type of AEs [4] and occur in 14%–30% of surgical procedures [5, 6]. Half of these are considered preventable [7]. Nurses are central to preventing surgically related AEs, as nurses make up a large proportion of postoperative care personnel [8]. The prevailing strategy for combating AEs is to anticipate them by standardising processes, protocols and checklists and to prevent them by learning from AEs (Safety-I) [9]. However, not all AEs can be anticipated or prevented. Patients have different risks and respond differently to therapy, and the healthcare system’s complexity is increasing [10]. Because of this complexity, healthcare has transitioned into the ‘age of safety management’, where humans are seen as an asset for safety in complex systems [11, 12]. This necessitates considerable integration of Safety-II logic to increase patient safety and resilience [11]. Resilience is the capacity to ‘adapt to challenges and changes at different system levels, to maintain high quality care’ [13] p. 1. Safety-II emphasises healthcare professionals’ adaptability in complex, ever-changing environments and focuses on learning from how work usually succeeds to prevent AEs [9]. The well-being of healthcare professionals is equally as important as performance in complex systems [14]. As a patient safety initiative that captures episodes of everyday successful work, it can be argued that Learning from Excellence (LfE) is a system for improving staff morale while ‘doing Safety-II’ [15, 16].

LfE was developed in Birmingham in 2014 and has since spread internationally, with positive local responses [17]. LfE’s main objectives are (1) to provide positive feedback to healthcare professionals and (2) to learn from what works well [18]. The core of LfE is the voluntary reporting (LfE reports) of a peer’s excellent work [18]. The LfE report is then forwarded to the identified positive deviant(s), thereby providing positive feedback. Selected LfE reports are explored with the positive deviant(s), using appreciative inquiry [19]. This conversation extracts valuable insights that can be disseminated and learned from [18]. Kelly et al. states that, hypothetically, ‘reporting and studying success would augment learning, enhance patient outcomes and experience through quality improvement work and positively impact resilience and culture in the workplace’ [18] p. 789. This points to organisational resilience, although individual resilience underpin the resilience of the organisations [12]. The hypothesis describes a patient safety culture (PSC), which can be defined as a group’s ‘behaviour, based upon shared beliefs and values that continuously seeks to minimise patient harm’ [20]p. 4. PSC can be divided into tangible measurable dimensions, such as teamwork climate, safety climate, working environment and healthcare professionals’ work engagement [21]. Significant variation in postoperative deaths between hospitals may be explained by differences in PSC [22–24]. Therefore, improving resilience and PSC are key pillars in the global strategy for eliminating preventable AEs [25]. In the United Kingdom National Health Service (NHS), healthcare professionals’ local responses to LfE have been ‘overwhelmingly positive’ [17] p.1. This has been reflected in cross-sectional studies based on surveys [18, 26], mixed methods studies [17, 27], quality improvement projects [28, 29], and to some extent, qualitative studies [30, 31]. Also, a LfE programme theory and a positive psychology (PP) logic model has been developed that suggest that LfE can positively impact the PSC [17, 32]. However, whether LfE has an effect on the tangible dimensions of PSC has not yet been evaluated.

PSC is a group-level property, and local efforts are necessary to improve it [33]. Hence, a postanaesthesia care unit (PACU) at a Norwegian university hospital, which is the context of this study, implemented LfE in November 2022. The PACU nurses wanted to balance learning from errors, using the Green Cross method (Safety-I), with learning from successful work (Safety-II) [34]. The Green Cross method is a simple visual method for reporting risks or AEs based on teamwork and safety briefings [34]. This study is the second part of an LfE evaluation in the PACU. Focus group interviews were conducted to describe healthcare professionals’ experiences of this complex intervention on the intangible dimensions of PSC [35, 36]. The results are reported elsewhere [31]. This study used an annual ‘Hospital Staff Survey’ to measure the tangible dimensions of PSC over a 16-month period (between 2023 and 2024) [35, 37].

This study aimed to explore whether the implementation of LfE in a postanaesthesia care unit over a 16-month period changed the nurses’ perceptions of the measurable PSC dimensions ‘work engagement’, ‘teamwork climate’, ‘working conditions’ and ‘safety climate’.

### Conceptual framework

**Fig. 1:**
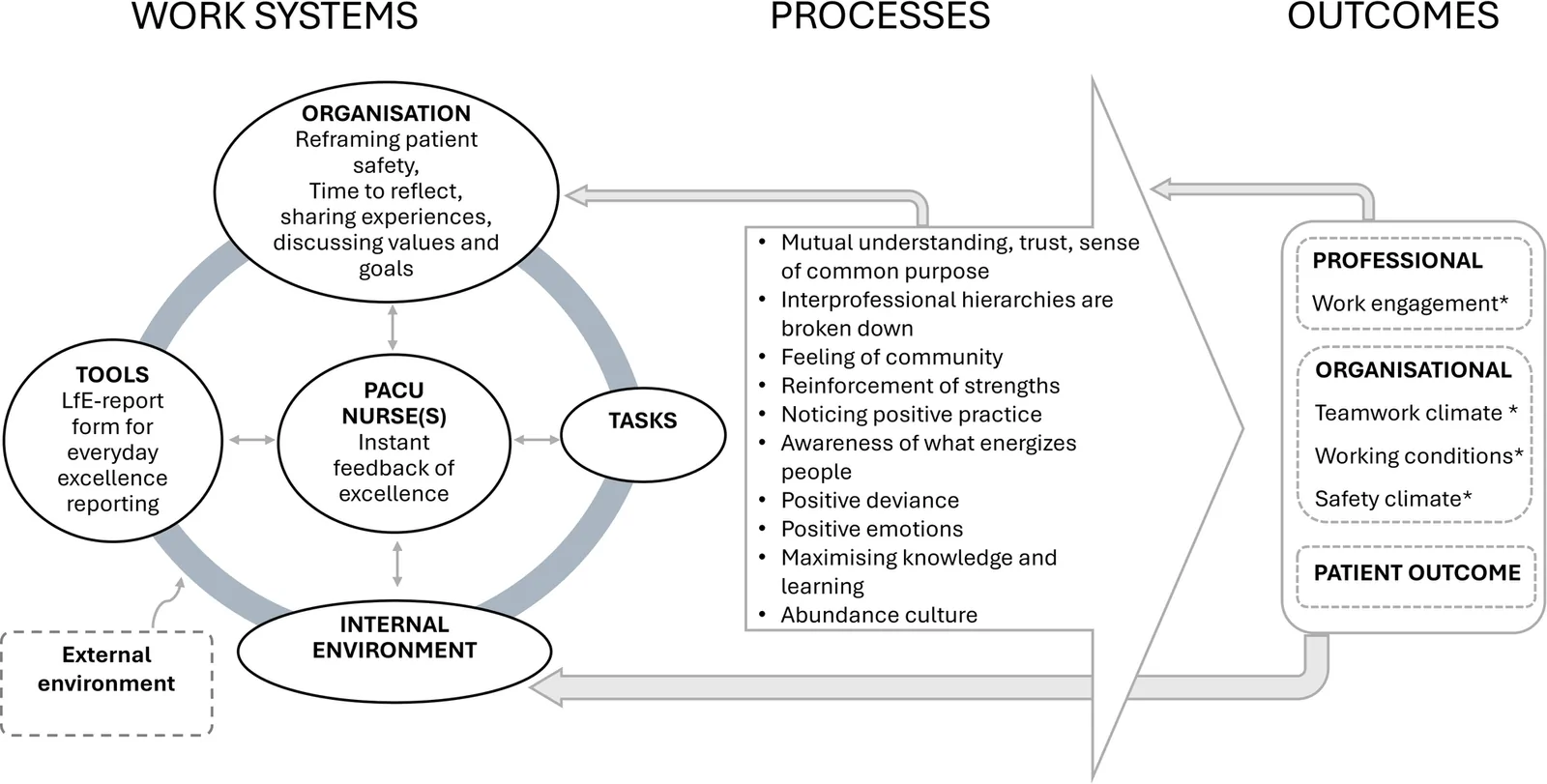
Modified SEIPS 101 model [39] This illustrates the study’s input and output and the complexity surrounding and influencing the intervention. The interrelatedness is marked by arrows. LfE=Learning from Excellence, PACU=postanaesthesia care unit ^*^Factors measured in this study

The conceptual framework used in this study describes how the LfE intervention (input) can impact work processes and shape outcomes (Fig. 1). This builds on the Systems Engineering Initiative for Patient Safety (SEIPS) model [38, 39], the LfE programme theory [17] and a PP logic model [32]. The LfE programme theory was developed in the UK using a survey (27% response rate) and case studies [17]. The PP logic model was developed using a systematic review of positive psychology interventions, such as LfE [32]. The models describe contextual factors and mechanisms leading to potential positive LfE organisational outcomes or LfE-like interventions [17, 32]. Reframing patient safety, that is, noticing a peer’s excellent work that led to safer patient care, reporting this, and trying to adjust so that work processes better promote successful work, can impact group level and individual outcomes positively.

Work systems consist of six components that interact simultaneously. People (as one of the components), here represented by the PACU nurses, are placed in the centre, symbolising that work systems should support people, not the other way around. The PACU nurses interact with the other five components: the Organisation, Tasks, Tools and Technologies and the Internal and External environment. In our study, LfE was an input in the Organisation component because it attempts to reframe patient safety by sharing examples of excellence [16, 17, 32]. The LfE report form was an input in the Tools component because it adds a new tool for reporting excellence via a quick response code. The combined work systems influence processes, which is clinical work in the PACU. This, in turn, influences professional, individual-level outcomes (work engagement) and organisational, group-level outcomes (teamwork climate, safety climate and working conditions). The feedback loops (arrows) represent adjustments over time and illustrate the system’s complexity [38].

## Methods

### Design

This study followed a longitudinal quasi-experimental non-equivalent comparison group pretest–posttest design [40]. This is an effective alternative when randomised studies are not practical and where one unit receives the intervention and a similar available unit does not receive the intervention [41, 42]. A longitudinal perspective is useful for evaluating the sustainability of an intervention [41]. The study report followed the Transparent Reporting of Evaluations with Non-randomized Designs (TREND) guidelines [43] (Supplementary Material 4).

### Setting and sample

The intervention unit was a PACU that had implemented the Green Cross method in 2019 ahead of the LfE implementation [34]. In Norway, a PACU is regarded as the lowest of three categories of intensive care units (ICUs) [44]. A PACU has equipment for the monitoring and treatment of patients with organ failure and employ nurses with expertise in intensive medicine [45]. The comparison unit was one of the hospital’s two surgical ICUs. It implemented the Green Cross method in 2018; however, it did not implement LfE. This unit was selected based on its similarities with the intervention unit [42], that is, based on the PACUs close relation to ICUs. However, the PACU had different in-patient acuity level and different nurses’ education level (Table 1).

**Table 1:** Profiles of the intervention and comparison units

Variable	Intervention unit: PACU	Comparison unit: ICU
Division/department	Surgical/anaesthesia	
Managers	Same managers 2021–2024*	
Operation	24 h a day, 7 days a week	
Incident monitoring	Hospital IRS and Green Cross method	
Age of patients	Paediatric to geriatric	
Number of beds	25	14
Number of patients treated per month	2021 = 815	2021 = 48
	2022 = 855	2022 = 40
	2023 = 876	2023 = 50
	2024 = 850	2024 = 45
Type of patients	General pre- and postoperative patients and overnight care for complex patients (intensive care stays^1)^	Intermediate and intensive care patients
Number of intensive care stays (mean days) per year	2021 = 455	2021 = 452
	2022 = 443	2022 = 354
	2023 = 310	2023 = 468
Length of intensive care stays (mean days)	2021 = 1.9	2021 = 8.6
	2022 = 1.3	2022 = 4.6
	2023 = 2	2023 = 5
Number of staff during the study period	77–81	118–141
Staff education (%): CCN/RN	50/50	90/10
Nurse: patient ratio	1:2–1:1	1:3–1:1

PACU = postanaesthesia care unit, ICU = intensive care unit, RN = registered nurse with at least two years of clinical experience, CCN = critical care nurse (a registered nurse with a clinical postgraduate education, with or without a master’s degree); IRS= incident reporting system. * Continued management throughout the study period in both groups; ^1^‘Intensive care stays’ includes a minimum of one of the following criteria: (1) duration of stay over 24 h from medical reasons, (2) mechanical ventilation, (3) death before 24 h, (4) transfer to ICU before 24 h and (5) medicinal infusion for the treatment of circulatory failure lasting at least 6 h

## Intervention

LfE was initiated by the PACU management and implemented in November 2022. The LfE team was responsible for planning and implementing LfE in the PACU, based on resources from https://learningfromexcellence.com/. This also included mini-appreciative inquiry questions (Supplementary Material 1). Kotter’s change model guided LfE implementation in a stepwise fashion [46]:

### Preparation phase

In the *Preparation phase*, (1) a sense of urgency was created. The PACU nurses wanted to learn from the successes as well as errors; (2) an LfE team was established, including a nurse manager, two anaesthesiologists, two PACU nurses, an IT consultant, a patient representative and an external consultant (first author) and (3) LfE was agreed to be used.

### Action phase

In the *Action phase*, 4) the vision and rationale were explained to the PACU nurses through a 15-minute presentation, in a staff meeting and through e-mails and posters; 5) LfE resources were provided to empower the intervention; 6) small wins were celebrated, i.e., the number of reports and improvement project results and 7) the LfE team continued to press on.

### To make it stick

*To make it stick*, 8) positive feedback was forwarded to positive deviant(s). The events were displayed on posters and in monthly bulletins to PACU nurses. Monthly reflexive meetings were planned to discuss successful events. Table 2 shows how LfE was used in the PACU. More details about the intervention are reported elsewhere [31].

**Table 2:** Learning from Excellence–as implemented in the intervention unit

Variable	What is done:	How it is done:
REPORTING	QR^2^ codes are displayed on posters at the premises. Voluntary reporting (using mobile phones) of peer excellence There are no restrictions or guides for the types of episodes that can be reported.	1. Who did something excellent? 2. What was excellent? 3. What can we learn from this? 4. What can we do to make this happen more frequently? 5. I work here (name allowed voluntarily). 6. I am a patient/next of kin (no name allowed).
FEEDBACK	Reporters receive a text message (acknowledgement)	‘Thank you for assigning this report to a colleague. The person in question will receive information of what you found excellent, and the episode will be used in work on improvement’.
	Recipients of LfE^1^ reports receive a text message notification from the nurse manager	‘Hello [name]! You have received an LfE^1^ report from [name]. [Insert text from the original report]. Excellent work! Sincerely, [name]’
LEARN	A one-hour meeting is held every other week during which, 1) the LfE^1^ team decides on which two LfE^1^ reports to explore in-depth based on reading/reviewing/discussing the latest LfE^1^ reports. 2) a nurse initiates an informal mini-appreciative inquiry^3^ to explore the event	Mini appreciative inquiry^3^ is either a group dialogue between those submitting and receiving an LfE^1^ report or an individual interview with either of them if the former is not possible. The duration is approximately five minutes. This includes the following: • **Definition**: Purpose of meeting–offering congratulations (acknowledging) • **Discovery**: Exploring what was excellent in this episode • **Dream**: What needs to be done to make this happen more often? (measures) • **Design**: How can we promote and share this excellent practice?
	Information from the mini-appreciative inquiry^3^ comprises ‘LfE^1^ of the week’.	Information from the event is written in a book and read aloud before each shift, three times a day, for one week for inspiration, learning and changing of culture. However, after six months, this stops because of feedback from PACU^4^ nurses. Instead, a monthly LfE^1^ meeting is facilitated, starting from late fall 2023/winter 2024, during which successful work is discussed by PACU^4^ nurses (30 min). This is facilitated by the LfE^1^ team.
	Text from two LfE^1^ reports per week are displayed on the information screen in the PACU^4^ nurses´ break room.	Include lively pictures for inspiration and learning.
	A summary of LfE^1^ reports is displayed on a whiteboard in the hallway for inspiration.	This is done by the LfE^1^ team. The summary includes the number of LfE^1^ reports (statistics) and anonymised text from the original LfE^1^ reports in the following categories: professional work/innovation/initiative/communication/management/good teamwork.
	Selected LfE^1^ reports, chosen by the LfE^1^ team, become a focus point for work on improvement.	Measures are taken to improve work, e.g., mandatory pre-round meetings regarding all intensive care and intermediate patients.

^1^Learning from Excellence; ^2^ Quick response; ^3^ See Supplementary Material 1; ^4^ postanaesthesia care unit

### Survey

Tangible dimensions of PSC can be measured using questionnaires, such as the Safety Attitudes Questionnaire (SAQ) [21, 47]. The ‘Hospital Staff Survey’ has been used by all Norwegian hospitals since 2018 [37] as part of the government-mandated national patient safety programme [37, 48]. The survey comprises 41 statements (items) that are separated into nine thematic factors [37]. Some of the items are from the validated Norwegian translated SAQ, in which two of the factors, ‘teamwork climate’ and ‘safety climate’, are used nationally as PSC quality indicators [37, 47]. Other items are from the validated General Nordic Questionnaire for Psychological Social Factors at Work [49] and from the non-validated items regarding Health, Safety and Environment [50, 51] (Supplementary Material 2). However, any assessment of the internal validity of the ‘Hospital Staff Survey’ has not been completed.

### Binary variable; the ≥ 75 score

All items were scored on a 5-point Likert scale of agreement (1 = *strongly disagree* to 5 = *strongly agree*). The score for each item was converted from 1 to 5 to a 0–100 scale and then divided by the number of respondents to obtain aggregated data of the mean at the unit level. Accordingly, a positive response corresponds to a score of *≥* 75. The ‘Hospital Staff Survey’ reports that were made available to hospital units included the following: number of sent invitations; number of answers; answers in percent; mean scores for each factor; and percentage of staff with positive responses (*agree* or *strongly agree*), i.e., scores ≥ 75 [37]. The available data thus include the broad binary category of ‘percent with positive’ (*agree or strongly agree*) versus ‘percent with negative or neutral responses’ (*disagree strongly*,* disagree slightly*,* neutral*) on the factor level, and not the more subtle variations of all response options. The *≥* 75 score indicates consensus within the unit, which correlates with patient safety [52]. See Supplementary Material 3 for computing details.

For the purpose of this study, and according to the trial registration, the following four factors were included: ‘work engagement’, ‘teamwork climate’, ‘working conditions’ and ‘safety climate’. The included factors represent important dimensions of PSC [21]. The factors also relate to the aforementioned LfE programme theory [17] and PP logic model [32]. Table 3 shows the included factors, and how they relate to PSC dimensions and potential outcomes of LfE, thereby describing the rationale behind the included factors in this study. For more details regarding inclusion/exclusion of factors, see Supplementary Material 2.

**Table 3:** Included factors and items from the ‘Hospital Staff Survey’, related PSC^3^ dimensions and related outcomes in the PP^1^ logic model and LfE^2^ programme theory

*Factors* and items	Related PSC^3^ dimensions [21] *p*. 8	Related outcomes of LfE according to the PP^1^ logic model [32] LfE^2^ programme theory [17]
*WORK ENGAGEMENT* - My work tasks engage me. - I tell my friends that this is a good place to work. - I get to develop myself through work. - I receive adequate training and guidance to enable me to do a good job. - I receive constructive feedback on the work I do. - Overall, I am very satisfied working here.	**Well-being**: ‘Job satisfaction, burnout and other psychosocial factors’	*Attitudes* (Job satisfaction, morale, motivation, trust and work engagement) *Attrition* (Reduced likelihood of leaving) Burnout (Reduced stress, anxiety and depression) *Culture change* (A more positive atmosphere and improved morale) *Confidence* (Sense of competence and personal accomplishment) *Positive emotions* (Well-being and happiness)
*TEAMWORK CLIMATE* - Here, different professions work well together. - Collaboration with other units works well. - I get support and help from my colleagues when I need it. - Here, it is easy to ask when there is something I do not understand. - Here, it is easy to speak up about patient care problems.	**Teamwork and collaboration:** ‘Working together as a team and coordination of care among staff’ **Openness**: staff feeling comfortable expressing their issues or concerns and questioning behaviours	*Collaboration at work* (Inter-professional hierarchies are broken down, appreciation of others, common ground, sense of community and teamwork) *Interaction and support*
*WORKING CONDITIONS* - I have sufficient resources or tools to do my job. - My workload is reasonable. - I manage to take a break during the shift/working day.	**Resources and constraints**: Resources for safety, including staffing, equipment, lack of time and training	
*SAFETY CLIMATE* - I report deviations and incidents that may lead to harm or errors. - Here, it is safe to speak up about objectionable conditions. - We openly discuss the mistakes and incidents that occur in order to learn from them. - I am encouraged by my colleagues to report any patient safety concerns I may have. - Here, medical errors are handled appropriately. - I would feel safe being treated here as a patient.	**Safety systems**: e.g., reporting systems **Reporting and just culture**: ‘Willingness to report and a culture that does not assign blame’ **Learning and improvement**: ‘A focus on improving systems’	*Patient safety* *Strengths* (Knowledge, personal skills, insight and self-reflection) *Work practices* (Quality improvement projects)

^1^positive psychology, ^2^Learning from Excellence, ^3^patient safety culture

### Data collection

Reports from the ‘Hospital Staff Survey’ were collected from 2021 to 2024. In March (March 1st to April 1st) of the respective years, the link to the survey was distributed via text messages or work e-mails, allowing for a four-week response time. The implementation commenced in November 2022. This means that two surveys were distributed before the start of the implementation (2021, 2022) and two surveys were distributed during the implementation (2023, 2024). All hospital staff with > 1% employment relationships were invited to participate [37]. This inclusion criterion was set nationally and meant that all employees were invited to participate. This was a 10-minute survey that could be completed at work or at home. Managers sent reminders to the staff collectively via mail and text messages during the survey period.

### Statistical analysis

Logistic regression models were used to relate the time and unit membership variables to each of the endpoints [53]. The response variables used were *binarised* versions of the ‘Hospital Staff Survey’ responses, corresponding to whether individuals had provided positive responses (‘*agree*’ or ‘*strongly agree’*) to the relevant questions on the Likert scale. This threshold was chosen because the percentage of staff with positive responses ( i.e., scores ≥ 75) was the data summary accessible from the available resources (Supplementary Material 3).

The independent variable was the intervention unit (IU), and the covariates used were time and an interaction between the two. Time was encoded as a *binary* variable and thresholded as being *before* or *after* the intervention, as there were two time measurements before the intervention and two after the intervention. This addressed the main aim of the study, which was to test whether LfE influenced PSC.

The results were presented as effect sizes, standard errors and associated p-values of the trained logistic regression model. A significance threshold of *p* = 0.05 was used. The effect sizes emerged as log-odds ratios, such that a size of zero meant no effect. The absolute sizes were interpreted as follows: 0 < ‘small’ < 0.4 < ‘medium’ < 0.7 < ‘large’ < 1.4 < ‘very large’. The odds ratio in each group was the ratio of providing a positive answer rather than a negative answer in the group.

The ‘intercept’ variable represents the odds in the comparison unit before the intervention. The ‘time’ effect represents the variation in the endpoints before or after the intervention in the comparison unit. The ‘IU’ effect represents the odds ratio between the groups before the intervention, i.e., the average over 2021 and 2022. The interaction effect of ‘time: IU’ represents the variation observed in the intervention unit over the intervention time period, in addition to the other variation described. Consequently, the ‘time: IU’ interaction was considered the main result of interest, as this can be interpreted as the effect of the intervention, adjusted for the other variations observed with time and between the groups. Statistical analyses were performed using R version 4.3.2 [54].

## Results

### Descriptive results

**Fig. 2:**
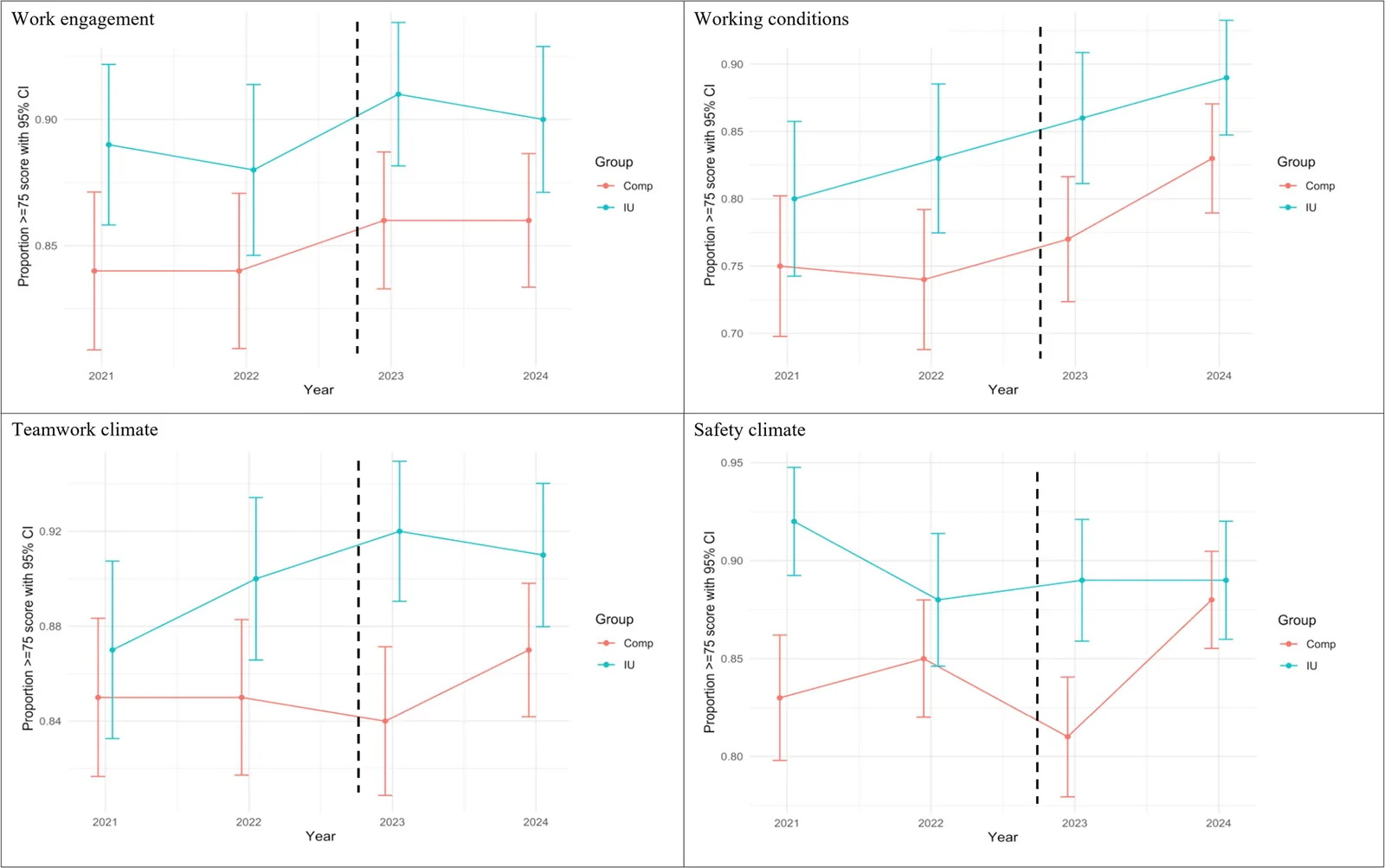
‘Work engagement’, ‘teamwork climate’, ‘working conditions’ and ‘safety climate’. Proportion of respondents in the intervention unit (IU) versus the comparison unit (Comp) that answered positively (≥ 75) on each factor. Dashed lines mark the start of the LfE intervention (November 2022)

Between 2021 and 2024, a total of 255 individual survey responses were completed in the intervention unit, with an average of 64 responses per year. A total of 394 individual survey responses were completed in the comparison unit, with an average of 99 responses per year. The response rates varied from 75% to 86% (mean = 81%) for the intervention unit and from 75% to 80% (mean = 77%) for the comparison unit. Figure 2 present the percentage of respondents in the intervention unit (IU) versus the comparison unit (Comp) who answered positively (≥ 75 score) on each factor.

### Logistic regression results

The results of the logistic regression analyses are presented in Table 4. The interaction ‘time: unit’ can be interpreted under some assumptions as the influence of the intervention, controlled for the other observed variation. The intervention had small positive effects on ‘work engagement’, ‘teamwork climate’ and ‘working conditions’, and a small negative effect on ‘safety climate’, although none of the effects were statistically significant. The groups were not well balanced before the intervention for any of the endpoints, with all outcomes being more positive in the intervention unit, although in principle this has been adjusted for when estimating the influence of the effect. Similarly, in the comparison unit, ‘working conditions’ increased significantly during the time period associated with the intervention; however, this has been included in the model as a background time effect.

**Table 4:** Results of the logistic regression analysis on patient safety culture factor scores

Factors	Work engagement	Teamwork climate	Working conditions	Safety climate
Intercept	1.658 (0.083)	1.735 (0.094)	1.072 (0.099)	1.659 (0.083)
	*p* < 0.001	*p* < 0.001	*p* < 0.001	*p* < 0.001
time	0.157 (0.116)	0.043 (0.128)	0.319 (0.140)	0.043 (0.113)
	*p* = 0.174	*p* = 0.739	*p* = 0.022	*p* = 0.707
IU	0.384 (0.143)	0.302 (0.158)	0.409 (0.167)	0.543 (0.149)
	*p* = 0.007	*p* = 0.056	*p* = 0.015	*p* < 0.001
time: IU	0.053 (0.203)	0.295 (0.227)	0.151 (0.246)	-0.155 (0.202)
	*p* = 0.793	*p* = 0.195	*p* = 0.540	*p* = 0.445

Results are presented as effect size (standard error) over p-values. ‘Intercept’ represents the odds ratio in the comparison unit before the intervention. ‘time’ represents time variation in the comparison unit, ‘IU’ represents the baseline difference between the groups before the intervention, and ‘time: IU’ represents the interaction of time and unit membership, i.e., the effect of the intervention

## Discussion

As related to the SEIPS model, there were no statistically significant improvements in any of the four PSC outcomes after LfE implementation. This is the first quantitative study to assess the effect of LfE on PSC. However, the results agree with the PP logic model [32]. The PP logic model was based on a systematic review of positive psychology interventions, in which only seven out of the 40 quantitative outcomes showed significant improvement, while the rest were neutral. In contrast, 12 of the 14 qualitatively assessed outcomes were positive [32]. Similarly, in the programme theory, all experiences with LfE were positive [17]. Interventions such as LfE tend to be evaluated more positively in qualitative studies than in quantitative studies [32, 55]. This suggests that there is a reporting or publication bias or that LfE may hold some value, despite the non-significant outcomes. However, effect estimates speak to the practical significance of the complex implementation [56], although they are inherently context bound and cannot predict effects in other units [36, 56]. This is discussed in the following paragraphs.

There was a non-significant and small (close to neutral) effect size increase in ‘work engagement’, which suggests that LfE had no significant or practical impact on the PACU nurses’ work engagement [56]. ‘Work engagement’ is a professional outcome in SEIPS, in which nurses’ well-being is regarded as equally important as performance [14, 38]. In this study, ‘work engagement’ includes, for example, ‘this is a good place to work’ and corresponds to the ‘well-being’ dimension of PSC [21] (Table 3). The result does not provide evidence to support the outcomes of LfE as described in the PP logic model and the LfE programme theory [17, 32], such as job satisfaction, motivation, improved morale and well-being (Table 3). Reframing negative interpretations may lead to ‘work engagement’ [16, 32]. The PACU nurses experienced that LfE facilitated a more positive work climate through reframing, which made them happy, energised and self-confident [31]. This agrees with British healthcare professionals’ experiences with LfE [30]. Simultaneously, the PACU nurses felt that LfE was potentially harmful, as those who never received LfE reports could feel unappreciated, jealous or left out [31]. This was partly why the practice of reading aloud information from a specific successful event three times a day was stopped after six months in the PACU (Table 2) [31]. This also agrees with British healthcare professionals’ experiences [30] and with those of Malawian community health workers [27], suggesting that this is a universal experience. This may be a possible explanation for the lack of a significant effect on ‘work engagement’. Psychological safety or ‘interpersonal risk climate’ has a direct impact on ‘work engagement’ [57]. LfE often emphasizes individual recognition. Valuing some members more than others may potentially unintentionally reduce psychological safety in the team [58, 59]. Although individual expertise is important, healthcare today is based on teamwork [60]. To ensure high-quality postoperative care and resilience, teams must excel at combining team members’ expertise [61, 62]. This is particularly true for hybrid teams within the PACU, which include permanent and rotating staff [63]. Focusing LfE reports more towards successful teamwork may be warranted in a PACU. Further studies should assess the role of psychological safety in LfE. The substantial differences in in-patient acuity level and nurses’ education level between the intervention group and the comparison group (Table 1) may have affected the outcomes in this specific factor score. Future quasi-experimental studies that explore the impact of LfE on ‘work engagement’ are advised to select a comparison group with same patient acuity score, nurse: patient ratio, and education level as the intervention unit.

‘Teamwork climate’ is regarded as an organisational outcome in SEIPS [38], and the results show a non-significant small positive effect on this factor. The large p-value does not support the evidence from the PP logic model that LfE improves collaboration, interaction and support (Table 3) [32]. This may be because increased interaction and support were the results of bringing people from different wards and units together to discuss and learn from each other [32]. This was not the case with the LfE intervention in this study; therefore, the result makes sense. Creating time and space for team reflexivity is the foundation of resilience in healthcare [64]. This enables a collaborative culture and shared understanding, which may break down inter-professional hierarchies and improve teamwork and quality improvement planning [65–67]. The small positive effect, however, may speak to the practical significance of this complex intervention [56]. A possible explanation is that the PACU nurses experienced LfE as a useful tool and a reminder to give more positive feedback between members of the hybrid team [31]. Gratitude cultivates social bonds and improves relationships and psychological safety, which are fundamental to building good teams [62, 68]. However, LfE reports were rarely reciprocated, since few units had implemented LfE [31]. Even if they were, it seems unlikely that random LfE reports could significantly improve the ‘teamwork climate’.

‘Working conditions’ are regarded as an organisational outcome in SEIPS [38]. The large p-value and the small effect size increase suggest that LfE had no significant effect on ‘working conditions’. However, the small increase was on top of the significant small effect size increase in the comparison unit. Thus, LfE may have had a small positive practical effect [56]. In this study, ‘working conditions’ included a reasonable workload and time to take a break (Table 3). This is an important PSC dimension [21] because the nurse work environment is a significant predictor of patient outcome [69]. However, this factor is not directly linked to outcomes described in the LfE model or the programme theory [17, 32]. It can be argued that improved collaboration, interaction and support can improve nurses’ perceptions of ‘working conditions’ [32]. The perfect hybrid team is adaptive and resilient, which necessitates fostering a culture of helping each other [62]. The PACU nurses felt that they helped each other more after LfE [31]. Additionally, they initiated an improvement project based on LfE reports that facilitated mandatory pre-round meetings for the sickest patients. This reduced the workload for all parties involved in the PACU [31]. These may be possible explanations for the small effect size increase. However, directed interventions to improve working conditions, such as adequate staffing, managerial support for nurses and good nurse-physician relations, are likely more effective than implementing LfE [69–71]. Consequently, we consider the most plausible explanations for the significant improvement in the comparison unit to be the increased staffing in 2023 and 2024 (from 121 to 132 and 141, respectively) and the implementation of the same staffing on weekends as on weekdays.

‘Safety climate’ is regarded as an organisational outcome in SEIPS [38]. The results show a non-significant, small negative effect on this factor. In this study, ‘safety climate’ includes items, such as ‘Here it is safe to speak up’ and ‘We discuss incidents to learn from them’ (Table 3). The negative effect size decrease in the intervention unit may be due to the high post-covid scores in 2021. The non-significant effect does not support the evidence that LfE increases patient safety [17], or that LfE augments learning [18]. A possible explanation is that in this intervention, there was no ‘sharing of experiences and history’ and no ‘time to reflect together’, which were commonly mentioned mechanisms in positive psychology interventions [32]. The PACU nurses felt that they did not acquire much knowledge by reading LfE posters [31]. However, they found it useful to discuss successful work in focus group interviews and suggested that this should be done regularly [31]. Accordingly, six months after implementing LfE, the PACU-nurses began 30-minutes monthly reflections on successful work (Table 2) [31]. Organisational learning is a social process that necessitates time for reflexive spaces [65, 66, 72], that is, ‘physical or virtual platforms in which reflexive dialogical practice occurs between people’ [64] p. 2. These spaces are considered key to patient safety [64]. Therefore, time set aside for reflexive spaces may improve ‘safety climate’ and ‘teamwork climate’, and this may be worth investigating in future LfE studies. Healthcare professionals find Safety-II-inspired reflexive spaces useful, but they find learning from successful situations difficult [73, 74]. In this study, monthly reflections were insufficient to have a significant effect on ‘safety climate’. As discussed above, LfE has a small tendency to emphasize individual contributions. The focus on individual excellence places significant responsibility on the LfE team and managers in how they act on LfE-reports. Complex systems require a systems perspective, and a work process evaluation could provide valuable insights into barriers and facilitators of successful work [75]. The implementation of LfE included the aforementioned improvement project [31]. While ‘quality improvement work’ agrees with the hypothesised effect of LfE [17, 18], one distinct improvement project was not enough to improve ‘safety climate’ in this study.

## Strengths and limitations

The study has several strengths. The longitudinal design made it possible to capture a richness of data and examine the sustainability of the effect of LfE in one setting over a 16-month period, which added to the external validity of the study [40]. Inference validity was strengthened by the use of two combined before and after measurements, which helped refute the regression to the mean as an alternative explanation [76]. Inference validity was further strengthened by the results, which agrees with previous qualitative findings in the PACU [31]. Internal validity was strengthened by the use of a comparison group, as this increased the ability to detect the effects of LfE and helped to partially control for confounders [41]. The ‘Hospital Staff Survey’ is used to indicate variation in PSC, at both local and national levels, and is thus a substantial source of information [37]. The annual survey reduced response attrition (75%–86% response rate), which could otherwise be a threat to internal validity. The use of the PP logic model and a conceptual framework to discuss the results strengthens the study’s construct validity, as does the use of Kotter’s steps to guide the intervention [46].

This study also has some limitations. *First*, the non-randomised design threatens the study’s internal validity, as causality is not inferred directly [41]. There were significant differences between the two groups in three of the four factor scores at baseline, suggesting that the two units were not well matched, although this was adjusted for when estimating the influence of the effect. Selection bias refers to the pre-existing differences between two groups that may affect the outcomes [40]. It was difficult to find a similar unit with the same nurse: patient ratio, the same nurses’ education level, and the same in-patient acuity level as the PACU, that had also implemented the GC method. In theory, a PACU from a different hospital that had implemented the GC method could have been used as comparison group. However, this would increase the bias risk of having differences in contextual factors that could affect the outcomes in the two groups. Also, it would be challenging to access the survey results from other hospitals. Because of the non-randomised design, the results could have been affected by other elements in the complex system, by secular trends that occurred at the same time or by contamination. This could have diminished the difference between the groups, weakening the power of the statistical analysis. PSC could have matured over time and been confused with the effect of LfE [38, 40]. *Second*, the ‘Hospital Staff Survey’ itself is not validated, although it contained questions from validated questionnaires. It does not include items regarding learning from successful work. This threatens the study’s construct validity, although the use of PP logic models helps mitigate this threat [40]. In addition, the high baseline scores may indicate a ceiling effect and leave little room for improvement due to a potential lack of sensitivity to the survey [40]. *Third*, the use of > 75 score as a dichotomous outcome (positive versus negative outcomes) is a very broad outcome. It would have been preferable to access more detailed responses. This could have strengthened the validity of the statistical inferences. However, these data were not available because secondary data were used. *Fourth*, the use of secondary data also precluded access to information about individual respondents. Although changes in the dependent variables were assumed to reflect changes in staff perceptions following the intervention, it is unknown whether the same individuals completed the survey at each time point. Consequently, the impact of staff turnover could not be assessed, and changes in PSC may partly reflect changes in workforce composition. However, the study focused on unit-level PSC rather than individual respondents. *Finally*, patient outcomes, such as AEs, were not measured. This was beyond the scope of this study but may be worth investigating in future LfE studies. Because of these study limitations, caution must be taken in generalising the results.

## Conclusion

This study shows no empirical evidence to support the effectiveness of LfE in changing PACU nurses’ perceptions of different factors associated with PSC over a 16-month period. The PSC factors ‘work engagement’, ‘teamwork climate’ and ‘working conditions’ had non-significant small effect size increases, whereas ‘safety climate’ had a non-significant small effect size decrease. We suggest that a systems perspective is important in enabling contextual conditions that enhance team resilience and adaptability. We suggest that future research focus on the role of psychological safety and well-being in LfE and assess whether reflexive spaces add value to LfE regarding learning. Given the time and effort spent on implementing LfE in healthcare, more longitudinal experimental studies are needed to assess its effect on PSC.

## Supplementary Information

Below is the link to the electronic supplementary material.

Supplementary Material 1

Supplementary Material 2

Supplementary Material 3

Supplementary Material 4

## Data Availability

The datasets used and/or analysed during the current study are available from the corresponding author on reasonable request.

## Abbreviations

AE: Adverse event
CCN: Critical care nurse
Comp: Comparison group
ICU: Intensive care unit
IU: Intervention unit
IRS: Incident reporting system
LfE: Learning from Excellence
PACU: Postanaesthesia care unit
PP: Positive psychology
PSC: patient safety culture
RN: Registered nurse
SEIPS: Systems Engineering Initiative for Patient Safety
TREND: Transparent Reporting of Evaluations with Non–randomized Designs
