# Supplementary material for: No effect of learning from excellence on patient safety culture in a postanaesthesia care unit: a longitudinal quasi-experimental study
Source: BMC Health Serv Res. 2026 Aug 26;26:1308. doi: 10.1186/s12913-026-15405-1 (PMC13613599; doi:10.1186/s12913-026-15405-1)
Supplement: Supplementary file 1 [file 12913_2026_15405_MOESM1_ESM.pdf]

Supplementary Material 1

Mini appreciative Inquiry. Used with permission from LfE.
https://learningfromexcellence.com/resources-and-evidence/resources/

LfE report number	
Subject of report/ role	
Participants	
Date of interview	
**Definition:**<br>Purpose of the meeting	
**Discovery:**<br>Please describe what happened that day?<br>What was it about you &/ or the team that made it happen?<br>What were the challenges and what techniques did you use to overcome them?<br>How did it feel?	
**Dream:**<br>Imagine, in 2 years' time, that situation is routine. What has changed?<br>What are the smallest steps we could take to make that happen?	
**Design/ Destiny:**<br>How can we promote and share this excellent practice?	
**Time Taken:**	
