# Supplementary material for: No effect of learning from excellence on patient safety culture in a postanaesthesia care unit: a longitudinal quasi-experimental study
Source: BMC Health Serv Res. 2026 Aug 26;26:1308. doi: 10.1186/s12913-026-15405-1 (PMC13613599; doi:10.1186/s12913-026-15405-1)
Supplement: Supplementary file 2 [file 12913_2026_15405_MOESM2_ESM.pdf]

Supplementary Material 2

Table. Included and excluded factors from the *'Hospital staff survey'* [1].

STAFF SURVEY FACTORS		STAFF SURVEY ITEMS	RELATED OUTCOMES AND ITEMS			
**Factors**	**Reasons for excluding or including factors**	**Translated to English** (by the authors)	**PP[1] logic model** [2] A **LfE programme theory** [3] B	**PSC[2] dimensions** [4]	**SAQ[3]** [5]	**QPS[4] Nordic** [6]
**I. Work engagement**	**Included.**<br><br>The items relate to LfE and PSC, and are part of SAQ. There are no elements that hinders its use.	1. My work tasks engage me	**Attitudes** as result of work: job satisfaction, morale, motivation, trust, work engagement. A + B<br>**Attrition:** reduced likelihood of leaving. A + B<br>**Burnout**: reduced stress, anxiety, and depression. A<br>**Confidence:** sense of competence, personal accomplishment. A<br>**Culture change**: a more positive atmosphere, improved morale. B	**Well-being**<br><br>"Job satisfaction, burnout and other psychosocial factors", page 8	**#19**. Morale in this clinical area is high.	**#27.** Is your work challenging in a positive way?
		2. I tell my friends that this is a good place to work			**#17**. This is a good place to work.	
		3. I get to develop myself through work				**# 86.** Does your immediate superior help you develop your skills?
		4. I receive adequate training and guidance to be able do a good job			**# 32.** Trainees in my discipline are adequately supervised.	**#25** Do you perform work tasks for which you need more training?
		5. I receive constructive feedback on the work I do			**#10.** I receive appropriate feedback about my performance.	

		6. Overall, I am very satisfied with working here	**Positive emotions**: well-being, happiness. A + B		# **15**. I like my job.	
**II. Teamwork climate**	**Included.**<br><br>The items relate to LfE and PSC, and are part of SAQ. There are no elements that hinders its use.	1. Here, different professions work well together	**Collaboration** at work: Interprofessional hierarchies are broken down, appreciation of others, common ground, sense of community, teamworking. A<br><br>**Interaction and support** A	**Teamwork and collaboration.**<br><br>**Openness:** “Open communication, staff feeling comfortable to express their issues or concerns and question behaviours”, page 8.	# **6.** The physicians and nurses here work together as a well-coordinated team.	
		2. Collaboration with other units work well.				
		3. I get support and help from my colleagues when I need it			# **4.** I have the support I need from other personnel to care for patients.	**#72** If needed, can you get support and help with your work from your coworkers?
		4. Here it is easy to ask when there is something I do not understand			# **5.** It is easy for personnel here to ask questions when there is something that they do not understand.	
		5. Here it is easy to speak up about problems in patient care			**#2.** In this clinical area, it is difficult to speak up if I perceive a problem with patient care.	
**III. Working conditions**	**Included.**<br><br>The items relate to PSC and (indirectly) to LfE, and are part of SAQ. There are no elements that hinders its use.	1. I have sufficient resources or tools to do my job		**Resources and constraints:** “Resources for safety including staffing, equipment, lack of time and training”, page 8.		**#42.** Are you given assignments without adequate resources to complete them?

		2. My workload is reasonable (number of tasks, pace of work or requirements to do several things at once)			**#29.** The levels of staffing in this clinical area are sufficient to handle the number of patients.	**#14.** Is it necessary to work at a rapid pace? **#15**. Do you have too much to do? **#47.** Can you set your own work pace?
		3. I manage to take a break during the shift/ working day				**#48:** Can you decide yourself when you are going to take a break?
**IV. Safety Climate**	**Included.**<br><br>The items relate to LfE and PSC, and are part of SAQ. There are no elements that hinders its use.	1. I report deviations and incidents that may lead to harm or errors	**Patient safety**: maximizing knowledge and learning. B<br><br>**Strengths:** knowledge and personal skills insight, self-reflection. A+B<br><br>**Work practices:** Quality improvement projects. A	**Safety systems:** "that facilitate patient safety (eg, rewards, reporting systems)", page 8.<br><br>**Reporting and just culture**: "Willingness to report and a culture that does not assign blame", page 8.<br><br>**Learning and improvement**: "A focus on learning from mistakes, responding to, and improving systems", page 8.	**# 9.** I know the proper channels to direct questions regarding patient safety in this clinical area.	
		2. Here it is safe to speak out about objectionable conditions			**# 11.** In this clinical area, it is difficult to discuss errors.	
		3. We openly discuss the mistakes and incidents that occur in order to learn from them			**#13**. The culture in this clinical area makes it easy to learn from the errors of others.	
		4. I am encouraged by my colleagues to report any patient safety concerns I may have			**# 12**. I am encouraged by my colleagues to report any patient safety concerns I may have.	
		5. Here medical errors are handled appropriately			**#8**. Medical errors are handled appropriately in this clinical area.	

		6. I would feel safe being treated here as a patient			**# 7**. I would feel safe being treated here as a patient.	
**V. Psychosocial work environment**	**Excluded**<br><br>Not directly relatable to the LfE or PSC.	1. Here, systematic work is done with the prevention and follow-up of violence and threats				
		2. I am not aware that anyone has been subjected to discrimination here in recent times 12 months				***31.** Have you been exposed to threats or violence at work during the last two years?<br>***81** Have you noticed anyone being subjected to harassment or bullying at your workplace during the last six months?
		3. I am not aware of anyone being sexually harassed here in the last 12 months				
		4. I am not aware of anyone being discriminated here in the last 12 months				
		5. When conflicts arise, they are handled in a good way			**#27a.** Problem personnel are dealt with constructively by our management (unit management).	
		6. I know how to proceed if I noticed that someone was subjected to bullying,				

		harassment or discrimination				
**VI. Perceptions of manage-ment**	**Excluded**<br><br>To protect anonymity. There is only one manager in the PACU.	1. My immediate manager has clear expectations of my work				
		2. My immediate manager is available to me when I need it				**#75.** If needed, is your immediate superior willing to listen to your work-related problems?
		3. My immediate manager follows up on adverse events and suggestions for improvement			**#14.** My suggestions about safety would be acted upon if I expressed them for management.	
		4. My immediate manager encourages me to speak up when I have a different opinion				**#85.** Does your immediate superior encourage you to speak up, when you have different opinions?
**VII. Perceptions of managing director**	**Excluded**<br><br>To protect anonymity.	The managing director supports the work with patient safety				
**VIII. Physical environment**	**Excluded**<br>Not directly relatable to LfE. The items mostly pertain to health, safety, and environment	1. There is a good physical working environment here (air, light, noise, dust, vibrations, etc.)			Health, safety and environment.	
		2. Here, we work well with fire protection				

		3. Here, the work is organized so that muscle and skeletal problems are prevented				
		4. Here, everyone is well protected against harmful chemicals and biological hazards				
		5. Here, we work well with infection control				
		6. Here, good work is done with radiation protection				
		7. Here, safety is well taken care of when using machines, technical aids or equipment				
		8. Here, good efforts are made to avoid negative impact on the external environment				
**IX. Follow-up**	**Excluded** The items pertain to follow-up on the survey responses in the unit. This has nothing to do with LfE.	1. Here, systematic work is being done to improve the working environment and patient safety				
		2. I have been involved in the follow-up of the previous Staff Survey				

[1] PP = Positive psychology. [2]PSC = Patient safety culture. [3]SAQ = Safety Attitudes Questionnaire. [4]QPS=Questionnaire for psychological and social factors at work

References:

1. The Norwegian Directorate of Health. ForBedring - kartlegging av sikkerhetskultur i spesialisthelsetjenesten [ForBedring - mapping of safety culture in the specialist health services]. 2017. https://www.itryggehender24-7.no/aktuelt/nyheter/enige-om-ny-hms-kulturundersokelse/_/attachment/download/01a23ae6-bad8-4c04-b4cc-c51dd0d0eb48:9528e684938e5d56669081adf2314c10a48fa983/forbedring-kartlegging-av-sikkerhetskultur-i-spesialisthelsetjenesten.pdf (accessed 3 March 2024).
2. Kletter M, Harris B, Brown C. Outcomes, mechanisms and contextual factors of positive psychology interventions for health workers: a systematic review of global evidence. Hum Resour Health 2021;19(1):24-. https://doi.org/10.1186/s12960-021-00564-5
3. Kletter M, Plunkett E, Plunkett A, Hunt H, Bird P, Brown C. Impact of the ‘Learning from Excellence' programme in NHS trusts: an exploratory study. Br J Health Care Manag 2020;26(8):1–10. https://doi.org/10.12968/bjhc.2020.0003
4. Churruca K, Ellis LA, Pomare C, Hogden A, Bierbaum M, Long JC, et al. Dimensions of safety culture: a systematic review of quantitative, qualitative and mixed methods for assessing safety culture in hospitals. BMJ open 2021;11(7):e043982. https://doi.org/10.1136/bmjopen-2020-043982
5. Deilkås ET, Hofoss D. Psychometric properties of the Norwegian version of the Safety Attitudes Questionnaire (SAQ), Generic version (Short Form 2006). BMC Health Serv Res 2008;8(1):191. https://doi.org/10.1186/1472-6963-8-191
6. Lindström K, Lindström K., Elo AL, Skogstad A, Dallner M, Gamberale F, et al. User's guide for the QPSNordic : general Nordic questionnaire for psychological and social factors at work. Copenhagen: Nordic Council of Ministers; 2000.
