# Supplementary material for: No effect of learning from excellence on patient safety culture in a postanaesthesia care unit: a longitudinal quasi-experimental study
Source: BMC Health Serv Res. 2026 Aug 26;26:1308. doi: 10.1186/s12913-026-15405-1 (PMC13613599; doi:10.1186/s12913-026-15405-1)
Supplement: Supplementary file 3 [file 12913_2026_15405_MOESM3_ESM.pdf]

Calculations of unit level factor scores in the Hospital staff survey 'ForBedring' 2021-2024

1. **The mean thematic factor score for each respondent:**

Likert scale	Response	Score
1	Disagree strongly	0
2	Disagree slightly	25
3	Neutral	50
4	Agree slightly	75
5	Agree strongly	100
6	Do not know/ not relevant	Removed from calculations

If one factor contain five items, and if one respondent responds: 3, 4, 4, 3, 4, the calculations would be: 50 + 75+ 75+ 50 + 75 / 5 (divided by five items for this factor)
mean factor score for this respondent = 65

If one factor contain five items, and if one respondent responds: 3,4,4,3,6, the calculations would be: 50+75+75+50 / 4 (divided by four because 'do not know/not relevant' is removed from the calculations)
mean factor score for this respondent = 62,5

2. **The mean thematic factor score for each unit/ward:**

Example 3: The calculations for the mean factor score of a unit with five respondents may look like this (the factor includes five items):

**Unit X**	Item 1	Item 2	Item 3	Item 4	Item 5	Divided by	(Mean)
Respondent 1	100	75	50	50	100	/5	75
Respondent 2	100	25	100	100	100	/5	85
Respondent 3	25					/1	25
Respondent 4	50	50	75	0	25	/5	40
Respondent 5	100	75	25			/3	67

Mean factor score for Unit X is:(75+85+25+40+67 / 5 respondents) = 58,4

3. The ≥75 score

The ≥75 score is the proportion (in percent) of employees who have answered 'Agree slightly' or 'Agree strongly' for all valid responses, that is, excluding the ´Do not know/not relevant' options. For the same responses as before in the same Unit X, the calculations of the ≥75 score for one factor will look like this:

**Unit X**	Item 1	Item 2	Item 3	Item 4	Item 5
Respondent 1	100	75	50	50	100
Respondent 2	100	25	100	100	100
Respondent 3	25				
Respondent 4	50	50	75	0	25
Respondent 5	100	75	25		

The green colour marks positive responses, whereas red colour marks neutral or negative responses. As visualized in the table above, there are 10 positive (green) responses, and 9 negative/neutral (red) responses in this factor. This makes 19 valid responses in total, as the white colour marks ´do not know/ not relevant' responses which are excluded from this calculation.

The >75 score of the Unit X is therefore: 10/19*100 = 53%
